# Minimally invasive comprehensive treatment for granulomatous lobular mastitis

**DOI:** 10.1186/s12893-020-00696-w

**Published:** 2020-02-22

**Authors:** Yaohuai Wang, Junlong Song, Yi Tu, Chuang Chen, Shengrong Sun

**Affiliations:** Department of Breast and Thyroid Surgery, Renmin Hospital of Wuhan University, No. 238 Jiefang Road, No.99 Zhang Road, Wuchang District, Wuhan, 430060 China

**Keywords:** Granulomatous lobular mastitis, Minimally invasive comprehensive treatment, Recurrence rate, Esthetic outcomes, Non-lactating mastitis

## Abstract

**Objective:**

To describe a minimally invasive comprehensive treatment for granulomatous lobular mastitis (GLM) and compare its effect with the existing methods, particularly in terms of its recurrence rate and esthetic outcomes.

**Methods:**

This retrospective study reviewed 69 GLM patients receiving the minimally invasive comprehensive treatment. Patients’ information, including age, clinical features, image characteristics, histopathological findings, mastitis history, treatment process, operative technique, recurrence, and esthetic effect, was evaluated.

**Results:**

All patients were female with a median age of 32 (range 17–55) years. Hospital stays ranged from 2 to 34 days, with a median of 6 days. The shortest time for complete rehabilitation was 2 days and the longest time was 365 days, with a median of 30 days. After a median follow-up of 391 days (range 162–690), 7 patients (10.14%) relapsed. The average cosmetic score was 2.62 ± 0.57 points and was mainly related to the past treatment, especially the surgical history.

**Conclusion:**

Minimally invasive comprehensive treatment is a new method for the treatment of GLM, ensuring a therapeutic effect while maintaining breast beauty.

## Introduction

Granulomatous lobular mastitis (GLM), first described by Kessler and Wolloch in 1972 [1], is non-lactating chronic mastitis that occurs primarily in women of childbearing age. The clinical and imaging features of GLM are very similar to those of breast carcinoma, resulting in many cases being misdiagnosed before the final pathological diagnosis. In the past, this disease was rare, but in the 30 years since the first report, about 200 cases of GLM were reported, mainly in Asian and Mediterranean countries, such as China, Iran, and Turkey [2, 3]. In recent years, the incidence of GLM has increased dramatically [4], possibly due to the increased awareness of GLM by clinicians or other reasons, which has aroused widespread concern from researchers around the world.

However, the definite etiology of GLM is unclear, but possible causes include hyperprolactinemia, microbial infection, and autoimmune disorders [5]. This uncertain etiology also leads to a lack of consensus on the optimal first-line treatment for GLM. Clinical observation, antibiotics, steroids, and surgery have been supported by different researchers, but surgery is the earliest and most widely used treatment. Surgical intervention has the advantages of short treatment time and low recurrence rate. However, surgery requires complete resection of the lesion and surrounding affected tissue; otherwise, healing of the incision will be difficulty and the recurrence rate will increase.

GLM often has a wide range of lesions, so the pursuit of a negative surgical margin can result in substantial surgical trauma and impair the beauty of the breast [6]. Therefore, nonsurgical managements, including antibiotics, steroids, and clinical observation only, are becoming more popular [7]. However, nonsurgical methods have drawbacks, including long treatment time, high recurrence rate, and potential side effects.

In this study, we described a minimally invasive comprehensive treatment for GLM and compared its effect with the existing methods, particularly with regard to the disease recurrence rate and esthetic outcomes.

## Materials and methods

### Study population

**Fig. 1:**
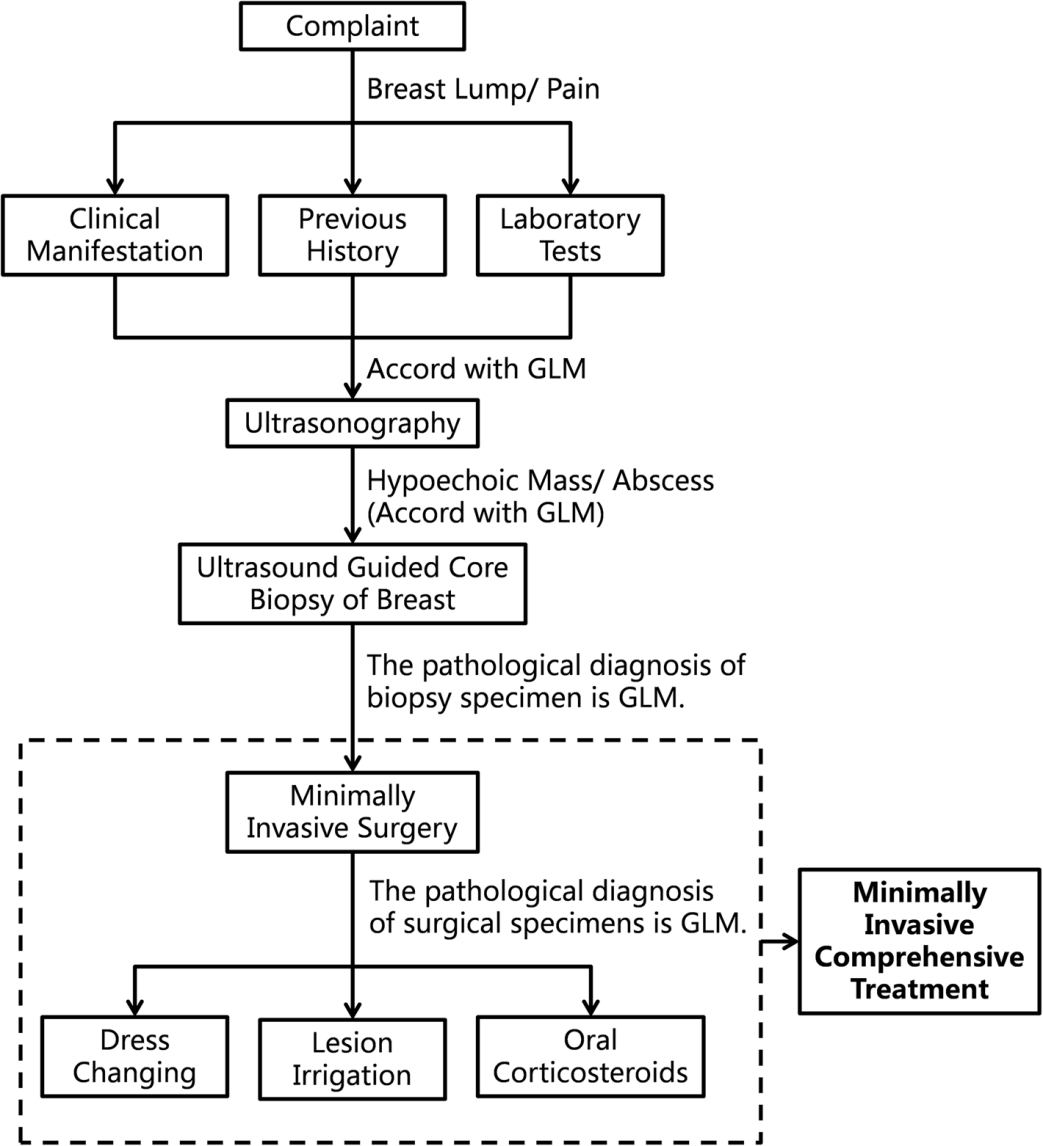
The workflow of minimally invasive comprehensive treatment

All GLM patients hospitalized in the Department of Mammary Surgery at the Renmin Hospital of Wuhan University from January 1, 2017, to December 31, 2017, were reviewed. The patients complained of “breast lump or pain”. The clinical manifestations, previous medical history, and laboratory examinations were consistent with the characteristics of GLM. The formation of hypoechoic masses or abscesses of breasts (according to GLM characteristics) was found under ultrasound. The ultrasound-guided breast needle biopsy was performed and sent for pathological examination. After the pathological diagnosis was GLM, minimally invasive surgery was performed. The specimens removed from the surgery were sent to routine pathological examination again. The pathological results were reviewed by two pathologists. After the diagnosis of GLM, dressing change, lesion washing, and oral steroid treatment were performed. Among them, minimally invasive surgery and subsequent dressing changes, lesion flushing and oral steroid therapy together constitute the “minimally invasive comprehensive treatment” described herein. The whole workflow is shown in Fig. 1.

Inclusion criteria: female; patients who were clinically diagnosed and pathologically confirmed with GLM; and patients undergoing minimally invasive comprehensive treatment.

Exclusion criteria: patients with tuberculous mastitis; patients with fat necrosis; patients with sarcoidosis; patients with periductal mastitis; patients with inflammation due to lactation or pregnancy; patients with other possible causes of mammary inflammation or granulomatous changes.

### Minimally invasive comprehensive treatment

All GLM patients received minimally invasive comprehensive treatment, which was operated with an ultrasound-guided Mammotome minimally invasive biopsy system. We placed the indwelling hoses in the lesion during the procedure and then flushed the lesion via those indwelling hoses. After the operation, we regularly changed the dressing and irrigated the lesion. The oral steroid was administered after confirming that the histopathology of the patient was found to be GLM. The specific treatment methods were as follows:

#### Minimally invasive operation

After determining the location, size, and extent of the lesion by ultrasound examination, the appropriate puncture points were selected. Local anesthesia was administered with 1% lidocaine and adrenaline to the tissues surrounding the puncture point, puncture path, and lesion. A small incision of about 30 mm long was made with a sharp blade at the puncture point. The Mammotome rotary scalpel was inserted through the skin incision into the bottom of the lesion under the guidance of ultrasound.

The rotary scalpel was then adjusted so that the groove was located behind the lesion and circumcision and aspiration were performed on the lesion. For patients with small lesions, complete excision was attempted. In patients with large lesions, many small pus cavities were often formed or merged into larger pus cavities. At this time, the focus of the minimally invasive operation was to try to open each pus cavity and expel internal pus, rather than pursuing a complete excision of the lesion to avoid causing greater damage to the patient’s breast structure. For lesions with complex internal structures, it was difficult to completely remove the pus, and we tried to insert a rotary scalpel from multiple puncture points for better surgical results.

#### Intraoperative placement of hoses and irrigation of lesions

**Fig. 2:**
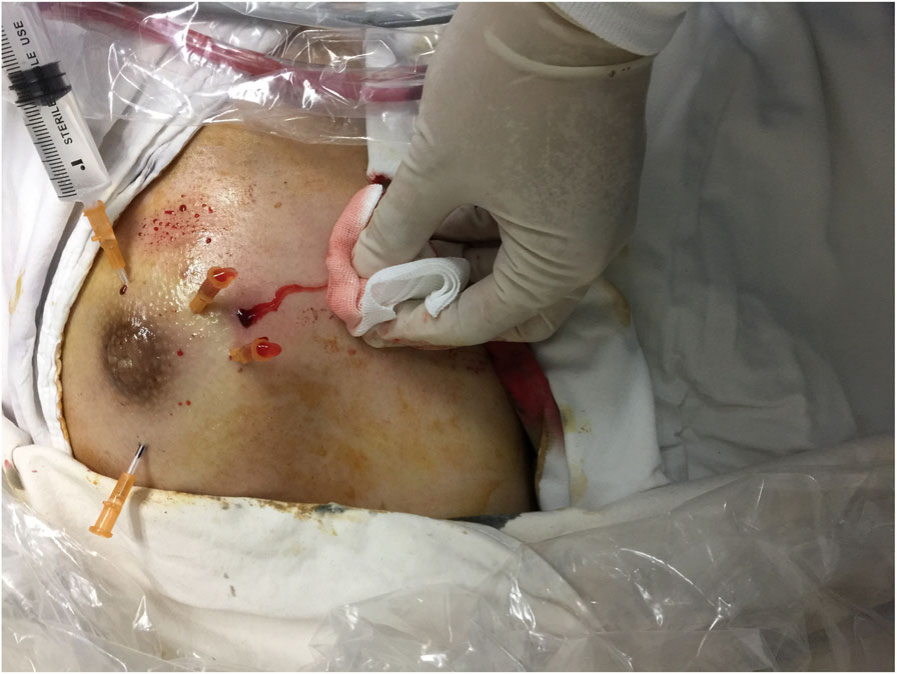
Irrigation of lesions. Normal saline was injected into the lesion through the indwelling hoses. Please note that liquid is flowing out of the other indwelling hoses and surgical inlets, indicating that the indwelling hoses are unobstructed

After excision of the lesion or opening of the pus cavity, the indwelling hoses were inserted into the lesion from the surface of the breast skin. The lengths and specifications of the indwelling hoses were selected according to the depth and scope of the lesion. Two to six indwelling hoses were used for unilateral breast lesions. The number of indwelling hoses could be increased appropriately for lesions with large scope and complicated internal structure, so as to achieve the effect of adequate drainage and flushing of the lesion. The hoses could also be inserted into the lesion from different directions to achieve a better irrigation effect. Normal saline was injected into the indwelling hoses to confirm that the hoses were unobstructed, and then the lesions were irrigated with hydrogen peroxide, metronidazole, and dexamethasone (Fig. 2).

#### Postoperative dressing change and lesion lavage

After the operation, each patient underwent regular dressing changes and lesion flushing. Here, the 2 to 6 indwelling hoses placed during surgery were used. One end of these hoses led to the lesion and the other was left on the skin surface. Because the lesion was the pus cavity or the surgical cavity remaining after the substantial lesion was surgically removed, so the lesion ends of the indwelling hoses were connected. When the drug was injected into the lesion from one indwelling hose, the drug can flow out of the other hoses, and the drug was continuously injected to play the effect of flushing the lesion. During rinsing, some pus, necrotic tissue, and newly generated pus that were not easily discharged in the cavity of the lesion can be flushed out of the lesion together, which was beneficial to the recovery and healing of the lesion.

Prior to every irrigation, normal saline was injected into the indwelling hoses to confirm the hose patency, and then the lesion was washed with iodophor, metronidazole, and dexamethasone. During the first week after surgery, dressing changes and irrigations were performed daily and then the frequency was gradually reduced according to the patient’s recovery. When the inflammation of the patient’s lesion subsided and no new pus was being produced, the irrigation fluid became clear, and the mass gradually softened and disappeared, at which point the indwelling hose can be gradually removed. All indwelling hoses were removed from 2 weeks to 1 month after surgery.

#### Oral steroid treatment

After each patient was histopathologically confirmed as GLM, oral prednisone was prescribed at a dose of 15 mg/day in the first week after surgery, 10 mg/day in the second week, and 5 mg/day in the third week and thereafter. The medication was discontinued when the patient was fully recovered or unacceptable side effects occurred.

### Data collection

Patients’ information, including age, clinical features, image characteristics, histopathological findings, mastitis history, treatment process, operative technique, recurrence, and esthetic effect, was analyzed. Disease recurrence was defined as the reappearance of a mass, abscess, or fistula on the ipsilateral breast. All patients were guided by an independent research nurse to evaluate cosmetic outcomes before and after minimally invasive comprehensive treatment using a semi-quantitative visual simulation scale (3 points, excellent; 2 points, good; 1 point, acceptable; and 0 points, poor), and all patients were divided into 2 groups according to whether they had a history of breast surgery in the hospital before this minimally invasive comprehensive treatment.

### Statistical analysis

Statistical analysis was performed with Statistical Product and Service Solutions 24.0 software. The results were reported as mean ± standard deviation for the continuous variables. A Wilcoxon rank-sum test for two independent samples was used to compare the differences in cosmetic scores between patients with or without previous breast surgical history.

## Results

### Ultrasound, mammography, and microscopy findings of all GLM patients

In 2017, 69 patients underwent minimally invasive comprehensive treatment in the Department of Mammary Surgery at the Renmin Hospital of Wuhan University. All patients ranged in age from 17 to 55 years, with a median age of 32 and an average age of 33.43 ± 7.36 years. Typical clinical features of GLM were breast masses or abscesses with or without overlying skin inflammation. In patients who had undergone previous abscess incision and drainage, sinus tracts formed by nonunion of surgical incisions were often seen.

Ultrasound findings of GLM were usually solid heterogeneous hypoechoic lesions with a diameter of 10–80 mm, irregular margins, and unclear demarcation from surrounding tissues (Table 1). Doppler ultrasound showed an increase in the number of blood vessels around the lesion. Axillary lymph node enlargement was observed in some patients. When an abscess had formed, the sonographic appeared as a cystic anechoic area with a large number of punctate debris echoes. The flow performance could be determined when the lesions were compressed. Mammography showed that the breast epidermis was thickened, with irregular nodules of increased density, and sometimes revealed sandy calcification or microcalcification. The ultrasound and mammography findings of GLM were similar to those of breast cancer and showed no specificity.

**Table 1:** General characteristics of patients (*n* = 69)

Characteristic	Number	Percentage (%)
Side		
Left	48	69.57
Right	21	30.43
Bilateral	0	0
Size		
< 1 cm	2	2.90
1–3 cm	21	30.43
3–5 cm	35	50.72
> 5 cm	11	15.94
Location (quadrant)		
Outer upper	9	13.04
Outer lower	8	11.59
Inner upper	8	11.59
Inner lower	5	7.25
Beneath areola	8	11.59
Involving 2 quadrants or more	31	44.93
Lesion Number		
Unifocal	57	82.61
Multifocal	12	17.39

Microscopically, GLM showed chronic suppurative granulomatous inflammation centered on terminal ductal lobular units. The lobule contained a large number of mixed inflammatory cell infiltration, mainly composed of neutrophils and sometimes plasma cells, but also included other lymphocytes, monocytes, and eosinophils. The lobules also showed non-necrotic non-caseous granuloma mixed with epithelioid tissue cells, foreign body giant cells, and Langhans giant cells. Small abscesses could also form in the granuloma. The acinar epithelium of the lobule atrophies disappeared or proliferated. When the lesions fused, the lobular structure disappeared, leaving large, patchy, and nodular chronic suppurative granulomatous lesions.

### Treatments and results in other hospitals

Of the 69 patients in the present study, 61 had a history of breast feeding, 13 had experienced milk stasis, and 6 had lactational mastitis. For the GLM event, 21 patients had received antibiotics, oral steroids, Chinese medicine, abscess incision and drainage (I&D), mastectomy, segmental mastectomy, or a combination of these methods in other hospitals. The antibiotics mainly included penicillin, levofloxacin, and cephalosporin. Some patients improved after treatment in the other hospitals, but no patient had a full recovery. The indicators of the patient’s condition improvement included signs of inflammation subsidence such as redness and swelling subsiding, pain reduction, lesion shrinkage, and sinus healing. See Table 2 for details of treatments and results in other hospitals.

**Table 2:** Detailed treatments and results from other hospitals

Treatment methods	Cases (n)	Improved Cases (n)
Antibiotics	5	3
Antibiotics + Oral steroids	1	1
Antibiotics + Traditional Chinese medicine	1	0
I&D^a^	2	1
Segmental mastectomy	2	2
Traditional Chinese medicine	1	0
Mass resection	5	2
Mass resection + Oral steroids	1	0
Mass resection + Antibiotics	1	0
Mass resection + I&D	1	1
Mass resection + I&D + Antibiotics	1	0
Total	21	10

^a^*I&D* incision and drainage

### Treatments and results in our hospital

After being admitted to the Department of Mammary Surgery at the Renmin Hospital of Wuhan University, all 69 patients underwent minimally invasive comprehensive treatment. After the inflammation subsided, the patients were discharged and continued to receive oral prednisone. The length of hospital stay was 2 to 34 days, with a median of 6 days and an average of 7.39 ± 4.89 days. After discharge, the patients attended the clinic for monthly reviews. If a physical examination found that the mass had disappeared, an ultrasound examination was performed. If the ultrasound confirmed the complete disappearance of the mass, the patient was considered to be fully recovered and the oral prednisone therapy was stopped.

During treatment, no patient developed intolerable steroid side effects. Thereafter, all patients were followed up every six months. The shortest time for complete rehabilitation was 2 days and the longest time was 365 days, with a median of 30 days and an average of 64.03 ± 78.36 days. After a median follow-up of 391 days (range 162–690), 7 patients (10.14%) relapsed after a few months. All patients with recurrence were treated again with minimally invasive comprehensive treatment and all recovered completely. There was no significant correlation between total hospital stay, complete recovery time, and disease relapse and previous treatment.

The average cosmetic score was 2.62 ± 0.57 points, which was mainly related to past treatment procedures, especially the history of surgery (including segmental mastectomy, mastectomy, and I&D). Patients with previous surgical history had an average cosmetic score of 2.15 ± 0.80 points, while patients without previous surgical history had an average score of 2.73 ± 0.45 points, and this difference was statistically significant (*P* = 0.006). Rank sum test was performed on the cosmetic scores before and after minimally invasive comprehensive treatment in three groups of patients: patients with previous surgical history, patients without previous surgical history, and all patients. The *P* values were found to be 0.020, 0.000, and 0.000, respectively, indicating that no matter whether the patient has a previous breast surgical history, minimally invasive comprehensive treatment can improve the cosmetic score of the breast (Table 3).

**Table 3:** Cosmetic scores of patients with or without previous surgical history

Cosmetic Score	Patients with previous surgical history (*n* = 13)		Patients without previous surgical history (*n* = 56)		All patients (*n* = 69)	
	Before MICT^a^	After MICT	Before MICT	After MICT	Before MICT	After MICT
Excellent (3 points)	2	5	12	41	12	46
Good (2 points)	2	5	17	15	18	20
Acceptable (1 points)	6	3	20	0	27	3
Poor (0 points)	3	0	7	0	12	0
*P*	0.020		0.000		0.000	

^a^*MICT* minimally invasive comprehensive treatment

## Discussion

At present, the primary treatment strategies for GLM include observation, antibiotic therapy, oral steroids, and surgery. No clinical consensus has been reached on the ideal therapeutic management, so both surgical and nonsurgical treatments have been advocated as the first-line treatments. Tables 4 and 5 show a review and summary of the representative studies on various treatment methods of GLM in recent years.

**Table 4:** Previous studies on various treatments methods for GLM

Author	Total			ATB^a^			Excision			I&D^b^		
	Treat	Recover	Recur	Treat	Recover	Recur	Treat	Recover	Recur	Treat	Recover	Recur
Li [8]	31	0 (0%)	NG^c^	31	0 (0%)	NG	–	–	–	–	–	–
Chirappapha et al. [9]	36	24 (66.67%)	NG	–	–	–	23	15 (65.22%)	NG	7	4 (57.14%)	NG
Shin et al. [10]	22	11 (50%)	5 (22.73%)	2	0 (0%)	–	20	11 (55%)	5 (25%)	–	–	–
Freeman et al. [11]	12	10 (83.33%)	2 (16.67%)	–	–	–	9	9 (100%)	2 (22.22%)	–	–	–
Yabanoğlu et al. [12]	77	77 (100%)	9 (11.69%)	–	–	–	33	33 (100%)	NG	–	–	–
Mahlab-Guri et al. [13]	8	7 (87.5%)	0 (0%)	2	2 (100%)	0	1	1 (100%)	0 (0%)	–	–	–
Korkut et al. [14]	73	60 (82.19%)	NG	–	–	–	36	32 (88.89%)	NG	37	28 (75.68%)	NG
Pandey et al. [15]	49	40 (81.63%)	10 (20.41%)	–	–	–	2	2 (100%)	0 (0%)	–	–	–
Karanlik et al. [16]	60	38 (63.33%)	7 (11.67%)	–	–	–	–	–	–	–	–	–
Al-Jarrah et al. [17]	20	18 (90%)	0 (0%)	19	17 (89.47%)	0 (0%)	1	1 (100%)	0 (0%)	–	–	–
Hovanessian Larsen et al. [18]	54	15 (27.78%)	NG	38	2 (5.26%)	NG	–	–	–	–	–	–
Lai et al. [19]	9	5 (55.56%)	0 (0%)	–	–	–	1	1 (100%)	0 (0%)	–	–	–
Total	451	313 (69.40%)	NG	92	21 (22.83%)	NG	126	105 (83.33%)	NG	44	32 (72.72%)	NG

^a^ATB, antibiotic therapy

^b^I&D, incision and drainage

^c^NG, not given

**Table 5:** Research reports on various treatments methods of GLM

Author	Observation			Steroid		
	Treat	Recover	Recur	Treat	Recover	Recur
Li [8]	–	–	–	–	–	–
Chirappapha et al. [9]	–	–	–	6	5 (83.33%)	1 (16.67%)
Shin et al. [10]	–	–	–	–	–	–
Freeman et al. [11]	–	–	–	3	1 (33.33%)	0 (0%)
Yabanoğlu et al. [12]	–	–	–	44	44 (100%)	9 (20.45%)
Mahlab-Guri et al. [13]	4	3 (75%)	0 (0%)	1	1 (100%)	0 (0%)
Korkut et al. [14]	–	–	–	–	–	–
Pandey et al. [15]	3	3 (100%)	0 (0%)	44	35 (79.55%)	10 (22.73%)
Karanlik et al. [16]	–	–	–	60	38 (63.33%)	7 (30.43%)
Al-Jarrah et al. [17]	–	–	–	–	–	–
Hovanessian Larsen et al. [18]	3	3 (100%)	0 (0%)	13	10 (76.92%)	NG
Lai et al. [19]	8	4 (50%)	0 (0%)	–	–	–
Total	18	13 (72.22%)	0 (0%)	171	134 (78.36%)	NG

*NG* not given

Some researchers believe that GLM is a self-limiting disease with good prognosis and suggest that expectant conservative management with close surveillance would be the treatment modality for GLM. For example, Lai et al. [19]. treated eight patients with expectant management with close regular surveillance and found that four patients (50%) had a spontaneous complete resolution of disease after a mean interval of 14.5 months. Those four patients did not relapse, whereas the remaining four patients had the static disease. Other reports have indicated that the healing rate of observation therapy for GLM can be even as high as 75–100% [13, 15, 18]. However, no large-scale studies have been conducted on observational therapy, and the results for cure rates and recurrence rates obtained at present are only applicable to patients with early diagnosis of mild illness. For patients who have developed abscesses or a large range of lesions, observation therapy often has little effect. In addition, when compared with other treatments, observation therapy has the longest average recovery time, causing patients with symptoms to endure long-term pain. In the present study, the median recovery time of GLM patients receiving minimally invasive comprehensive treatment was 30 days, and the longest recovery time was 1 year, which were both significantly lower compared with the observation therapy. The greatest advantage and attraction of observational therapy is that it does not require surgery or any medication. Nevertheless, because the clinical and imaging features of GLM are very similar to those of breast carcinoma, tissue biopsy remains the gold standard to confirm the diagnosis. Therefore, the choice of observation therapy does not necessarily avoid surgery. Besides, a minimally invasive operation has no significant difference in terms of surgical trauma and esthetic effects on the breast when compared with a core biopsy.

The clinical manifestations of GLM are similar to those of breast infections and abscesses, so antibiotics are usually used as empirical treatment. Hovanessian Larsen et al. administered an antibiotic treatment, including dicloxacillin, cephalexin, or clindamycin, to 38 patients for 10 days, but only two patients showed improvement [18]. Similarly, the preoperative antibiotic treatment used in 31 cases by Li, et al. [8]. was ineffective in 23 cases and none of the patients fully recovered; eight cases showed an effect of antibiotic treatment that was limited to reduction in lump and skin redness, suggesting that GLM may be complicated by pathogen infection and that the use of antibiotics helps to eliminate the relevant infection symptoms. On the contrary, Al-Jarrah et al. [17]. demonstrated good results with antibiotic therapy. All patients were managed conservatively with systemic antibiotics consisting of augmentin (1 mg twice a day) for 6 weeks and metronidazole (400 mg three times a day) for two weeks, and 17 out of 20 patients (85%) showed significant improvement. The difference in antibiotic efficacy between studies may be due to accidental or specific types of antibiotics used by researchers. Metronidazole used by Al-Jarrah et al. is a commonly used antibiotic in the clinic. It is mainly used for systemic or local infection caused by anaerobic bacteria, especially for Bacteroides, Clostridium, some Eubacteria, digestive cocci, and digestive Streptococcus. Some researchers believe that the occurrence of GLM is related to anaerobic bacteria, especially Corynebacterium [20, 21]. Based on this consideration, in our treatment design, we used hydrogen peroxide, iodophor, and metronidazole to irrigate the lesions during and after surgery, and we achieved good results.

GLM is usually characterized by chronic inflammation and can be observed by light microscopy. Some researchers believe that the pathogenesis of GLM may be an autoimmune response to the secretion of mammary ductal proteins, so they have attempted to use steroids to treat GLM and achieved some positive results. For example, Karanlik et al. [16] treated 60 patients with methylprednisolone and found the median response rate was 75% (25–100%) by observing the clinical and radiological regression in all patients receiving the steroid therapy; complete clinical and radiological regression was observed in 38 patients (63%). The major disadvantage of steroid therapy alone is its high recurrence rate. They reported that 7 of 23 patients who received steroid therapy only had recurrences, while no recurrence was observed in patients who underwent extensive resection after steroid treatment [16]. Another unsatisfactory aspect of steroid therapy is that the patient’s recovery time is longer. Yabanoğlu et al. [12]. recruited 77 patients, 33 of whom underwent surgery and 44 received steroid therapy. Recovery time was 6 (1–15) months in the steroid group while 1 (1–5) months in the surgery group (*p* = 0.001). In our study, the median recovery time after minimally invasive comprehensive treatment was 1 month, which was basically the same as the surgery group in the above study, but shorter than the steroid group. Besides, steroid therapy also has a notable problem that it may have side effects at high doses. Methylprednisolone was administered at a dose of 0.5 mg/kg/day for 4 weeks and four (7%) patients were reported to have a cushingoid appearance and hirsutism. In our study, the dose of steroids was relatively small and no related side effects were found. Immunosuppressive therapy is recommended for patients who have relapsed after steroid therapy and have steroid resistance or unbearable side effects [13]. However, due to the small number of reported cases, the therapeutic effect of immunosuppressants remains unclear.

Surgery has been one of the main treatments since GLM first reported. In terms of recurrence and post-treatment recovery, studies including surgical resection as a first-line treatment showed significantly superior results compared with steroid therapy alone [13]. Common surgical methods include I&D, mass resection, segmental mastectomy, and mastectomy. The biggest problem with surgical treatment is the contradiction between the surgical effect and the postoperative aesthetic effect. A reduction in the recurrence rate after surgery requires complete removal of the lesion and assurance of a negative margin, but achieving this often causes great damage to the breast structure. Some patients with large lesions even require a total mastectomy. Since GLM mainly occurs in women of childbearing age, breast loss is unacceptable in terms of aesthetics and breastfeeding. In our patients with large lesions, we did not completely remove the lesion with minimally invasive surgery but routinely flushed the lesion after surgery to reduce recurrence. In this way, we can guarantee the therapeutic effect while minimizing breast damage to patients and maintaining the beauty of the breast. In the previous literature, the recurrence rate of GLM after surgery was about 8–50% [12]. Of all the cases studied here, seven patients had recurrence with a recurrence rate of 10.14%, indicating that this was not significantly different from surgery. Of all 69 patients, 66 patients rated the cosmetic score of their breasts after treatment as excellent or good. None of our patients underwent a total mastectomy.

The minimally invasive comprehensive treatment described in this study mainly included three parts, namely minimally invasive surgery, postoperative dressing change, and lesion washing, and oral steroids. The main complication of surgery is the damage to the breast’s breastfeeding function and aesthetics, but the minimally invasive surgical method with less damage was used in this study, so the impact of this complication was small. There were no significant complications after dressing change and lesion washing. The main complications of oral steroids include weight gain, amenorrhea, osteoporosis, femoral head necrosis, Cushing’s syndrome, etc. There were no reported cases of the above-mentioned complications in this study. The possible reason was that, compared with the commonly used therapeutic dose (0.8 mg/kg/day) in other studies, the oral steroid dose in this study was smaller (15 mg/day) [22, 23].

Our research also has some limitations. It was a single-center study with a relatively short time span, and it was a retrospective study. Some treatments might have been changed due to the patient’s personal wishes, which may also have an impact on the results of the study.

## Conclusion

GLM is a nonspecific inflammatory disease that occurs in women of childbearing age and has no specificity in ultrasound and mammography results. Minimally invasive comprehensive treatment is a new treatment for GLM that can ensure a therapeutic effect while maintaining the beauty of the breast. Many problems still remain to be solved in the etiology, diagnosis, and treatment of GLM. Therefore, more in-depth large-scale, multi-center and prospective studies are needed.

## Data Availability

The data that support the findings of this study are available from the corresponding author upon reasonable request.

## Abbreviations

GLM: Granulomatous lobular mastitis
I&D: Incision and drainage
